# Enhanced recovery after colorectal surgery in patients with inflammatory bowel disease to reduce hospital length of stay: A systematic review and meta‐analysis

**DOI:** 10.1111/codi.70642

**Published:** 2026-09-29

**Authors:** Garrett Johnson, Evangelia Theodosopoulos, Jordyn Vernon, Selina Schmocker, Hilalion (San) Ahn, Javier Gomez, Mantaj S. Brar, Aman Pooni, Erin D. Kennedy

**Affiliations:** ^1^ Department of Surgery University of Toronto Toronto Ontario Canada; ^2^ Zane Cohen Centre for Digestive Diseases Mount Sinai Hospital Toronto Ontario Canada

**Keywords:** enhanced recovery after surgery, enhanced recovery program, fast‐track surgery

## Abstract

**Aim:**

Enhanced recovery after surgery (ERAS) improves outcomes in colorectal cancer surgery; however, patients with inflammatory bowel disease (IBD) have been relatively excluded from these analyses. The purpose of this systematic review was to evaluate the effects of ERAS protocols in adult patients with IBD.

**Methods:**

Relevant databases were searched for randomised‐controlled trials (RCTs) and controlled observational studies that assessed ERAS protocols versus conventional care in adult patients with IBD, in any language until July 21, 2025. Two authors independently identified trials, extracted data, assessed risk of bias (ROB 2.0 and ROBINS‐I) and evidence certainty (GRADE). Primary outcomes were length of stay (LOS), 30‐day readmissions, re‐operations and complications.

**Results:**

Eighteen studies (one RCT, 17 observational) were included. LOS was significantly lower in the ERAS group (mean difference: −1.62 days; 95% CI: −2.03, −1.21, 15 studies, 4225 patients, moderate certainty). There was no difference in 30‐day readmissions (odds ratio, OR: 0.83; 95% CI: 0.62, 1.12, 15 studies, 4157 patients, low certainty), 30‐day reoperations (OR: 0.68; 95% CI: 0.46, 1.02, 8 studies, 2161 patients, very low certainty), or 30‐day complications (OR 0.69; 95% CI: 0.45, 1.08, 14 studies, 4074 patients, low certainty). Evidence certainty was limited by non‐blinding, and observational data at serious risk of bias.

**Conclusions:**

ERAS for patients with IBD reduces LOS with no difference in 30‐day readmissions, reoperations and complications. Subgroup analyses generated the hypothesis that procedure‐specific ERAS pathways incorporating greater preoperative optimisation may provide additional benefit, but this requires confirmation in adequately powered randomised trials.

**What does this paper add to the literature?**

This systematic review with meta‐analysis summarises current evidence for enhanced recovery after colorectal surgery (ERAS) for inflammatory bowel disease (IBD). Based on one randomised trial and 17 observational studies, ERAS is associated with shorter length of stay and no differences in 30‐day readmissions, reoperations, or complications compared with conventional care.

## INTRODUCTION

Enhanced recovery after surgery (ERAS) is a multimodal perioperative approach that reduces surgical stress and promotes recovery. These standardised protocols aim to improve postoperative outcomes, including pain, bowel function, wound healing and hospital length of stay (LOS) [1]. While strong evidence shows that ERAS improves outcomes in colorectal cancer surgery [1, 2], data on its effects in inflammatory bowel disease (IBD) are more limited. ERAS may not confer the same benefits in IBD surgery because of differences in patient characteristics, disease course and surgical context [3]. Patients with IBD often present with malnutrition, systemic inflammation and immunosuppression, all of which can impair recovery [4]. Their surgeries are frequently urgent or emergent [5, 6], limiting preoperative optimisation measures, which are integral to ERAS [7]. IBD surgeries are also technically complex due to inflamed tissues disrupting normal planes and the increased rate of re‐operative surgery. Due to these factors, postoperative recovery may be further complicated by higher stoma rates, ileus, increased leak risk and pre‐existing chronic pain. Many patients with IBD have lower functional reserves and more variable recovery trajectories, with ongoing disease activity and continued medical therapy influencing outcomes [5, 6]. These factors may limit the effectiveness of standard ERAS protocols, which were designed for elective colorectal cancer populations [1]. Therefore, it is unclear how standard colorectal ERAS protocols apply or whether existing protocols need to be tailored to better support patients with IBD. This review aims to identify, critically appraise and summarise evidence on the efficacy and safety of ERAS protocols compared with conventional care in adults undergoing colorectal surgery for IBD.

## METHODS

A systematic review and meta‐analysis was conducted in accordance with the Preferred Reporting Items for Systematic Reviews and Meta‐Analyses (PRISMA) 2020 guidelines [8] and the Methodological expectations for Cochrane intervention reviews (MECIR) [9]. The protocol was prospectively registered with the International Prospective Register of Systematic Reviews (PROSPERO: CRD42022289701).

### Inclusion criteria

This review included randomised controlled trials (RCTs) and controlled observational studies evaluating ERAS in adult patients (≥18 years) undergoing colorectal surgery for IBD. Both published and unpublished trials were included. Uncontrolled observational studies, reports with insufficient data and duplicate reports were excluded. For duplicates, the latest and most complete reports took precedence.

We defined colorectal surgeries as those involving major abdominal surgery on the colon and/or rectum, including small bowel resections performed for Crohn's disease. Surgeries for the purpose of ileostomy reversal, or for isolated perianal disease were excluded. For studies where both included and excluded patient populations were presented (e.g., mixed paediatric and adult patients), ≥90% of the analysable population had to be from the included population.

We included studies evaluating a comprehensive ERAS protocol against a conventional non‐ERAS pathway. We defined ERAS as coordinated efforts to optimise various aspects of care, including preoperative, intraoperative and postoperative phases of care. An ERAS protocol had to describe a multimodal effort targeting ≥2 phases of care and include ERAS Society‐recommended elements [1]. Studies targeting only one phase of care were excluded (e.g., laparoscopic versus open surgery without other ERAS components). Non‐ERAS care was defined as perioperative care without a formal ERAS program.

### Outcomes

Primary outcomes were postoperative hospital LOS, 30‐day reoperations, 30‐day readmissions and total 30‐day complications. Secondary outcomes were ERAS protocol compliance and rates of individual complications [death, bleeding, ileus, anastomotic leak, urinary tract infection (UTI) and surgical site infections (SSI)] within 30 days of surgery. SSIs were classified according to CDC criteria [10].

### Data extraction

MEDLINE (Ovid), EMBASE (Ovid), Cochrane Central Register of Controlled Trials (Ovid EBM review), Cumulative Index to Nursing and Allied Health Literature (CINAHL) with Full Text (EBSCO), LILACS (VHL) and Web of Science Core Collection (Clarivate) were searched from inception until 21 July 2025 without language, publication year, or publication status restrictions. Detailed search strategies are shown in Supporting Information S1. Unpublished or ongoing clinical trials were identified by searching clinicaltrials.gov and the World Health Organization International Clinical Trials Registry Platform (who.int/trialsearch). Reference lists of included studies were also screened. Prior to publication, we searched the Retraction Watch Database (https://retractiondatabase.org) for included studies.

Two reviewers independently screened all titles and abstracts, assessed full‐text articles for study eligibility and inclusion and extracted data using standardised pilot‐tested forms. Included studies were assessed for risk of bias using methods suggested in the Cochrane Handbook for Systematic Reviews of Interventions [11]. The revised Cochrane risk‐of‐bias tool for randomised trials (RoB 2) was used for RCTs, and the ROBINS‐I V2 tool for observational studies [12, 13]. Evidence certainty for each outcome was evaluated according to the GRADE approach [14] using GRADEpro GDT software (www.gradepro.org). Differences between analysts were resolved through consensus.

### Statistical analysis

Data synthesis was conducted in Review Manager 5.4. A meta‐analysis was performed using a random‐effects model. Pooled continuous data were expressed as a mean difference (MD) with 95% confidence intervals (CI). Whenever possible, continuous data were transformed to the same scale to report MD. Pooled dichotomous data were presented as odds ratio (OR). For studies where more than one comparison was possible, we selected the condition most similar to other included comparator interventions. Where a study contained unclear information, its corresponding author was contacted. Data reported in non‐compatible form for meta‐analysis were imputed from available data if possible, as recommended [15, 16]. Because medians and interquartile ranges often indicate skewed data, sensitivity analyses were planned with these data excluded.

Publication bias was assessed by viewing confidence interval overlap and using funnel plots when ≥10 studies were included for an outcome [17]. We assessed heterogeneity through forest plot inspection, Chi^2^ and *I* ^2^ statistics [18], considering heterogeneity substantial when *I* ^2^ statistic was >40% [18]. Subgroup and sensitivity analyses were conducted to investigate heterogeneity for the primary outcomes. Subgroup analyses were planned for studies including patients with: ulcerative colitis (UC) versus Crohn's disease (CD); with and without new ostomies; type of surgical procedure (studies with ERAS intended for a single surgical procedure versus those designed for multiple surgical procedures); use of non‐steroidal anti‐inflammatory drug (NSAID); and ERAS protocol fidelity. High fidelity ERAS protocols (>50% compliance with all reported elements) were compared to those with low fidelity and those that did not report compliance, and further sensitivity analysis was done to look at the effect of specific ERAS components on primary outcomes. Sensitivity analyses were also planned to assess the robustness of the findings to key analytical assumptions, including exclusion of unpublished studies, studies with potential conflicts of interest, high/serious risk‐of‐bias studies, switching from random‐effects to fixed‐effect models, excluding imputed or potentially non‐normally distributed data, and by study design (RCT versus observational studies). Because only one randomised trial met the inclusion criteria, randomised and observational studies were pooled in the primary analyses to provide the most comprehensive estimate of treatment effect. Study design was incorporated into the assessment of certainty of evidence through separate risk‐of‐bias assessment and GRADE evaluation, and sensitivity analyses were undertaken to explore the influence of study design.

## RESULTS

### Study details

The systematic search yielded 1406 titles, including 1385 from bibliographic databases, 21 from trial registries and 3 from reference screening (Figure 1). After 321 duplicates were removed, 1088 studies were screened and 50 proceeded to full‐text review. One study was a clinical trial registry for an ongoing study with no available data [19], and 31 were excluded. The remaining 18 studies met full inclusion criteria. Two were published as abstracts [20, 21], and 16 were full text reports [3, 22, 23, 24, 25, 26, 27, 28, 29, 30, 31, 32, 33, 34, 35]. Two study authors responded to our query and provided additional unpublished data for analysis [3, 22]. Characteristics of included studies are summarised in Table 1. Detailed rationale for full text excluded studies are in Supporting Information S2. Details of the ongoing study are in Supporting Information S3.

**FIGURE 1:**
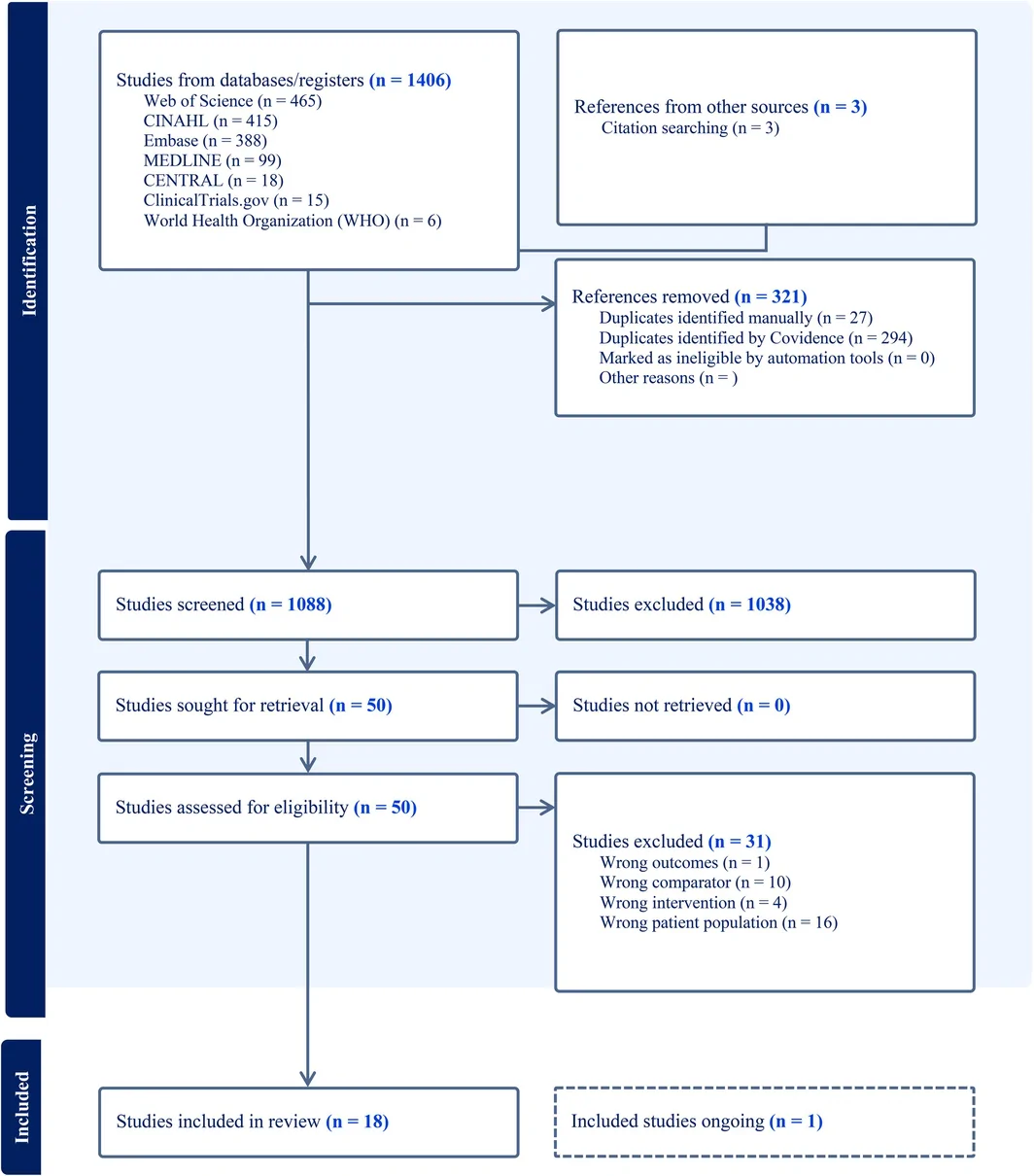
PRISMA flow diagram of included and excluded studies.

**TABLE 1:** Characteristics of Included Studies.

Study ID, country	Funding/conflicts	Study design	Disease type, n	Resection type, n	Operative characteristics, n	Relative effect of ERAS for primary outcomes (95% CI)
Aviran 2025 [22] USA	None	Retrospective observational open label (before/after study)	CD: 463 (48%), UC: 501 (52%)	Partial colectomy: 17 (2%) Pelvic anastomosis: 256 (27%) RHC/IC resection: 350 (36%) SBR: 377 (39%)	MIS: 457 (47%) Elective: 964 (100%)	LOS: MD −1.00 (−1.45 to −0.55) days Readmissions: OR 1.05 (0.74 to 1.49) Complications: OR 3.09 (2.03 to 4.70)
Chang 2017 [20] USA (abstract only)	Not reported	Retrospective observational open label (before/after study)	UC: 500 (100%)	CP + IPAA + ileostomy: 155 (28.2%). STC + ileostomy: 239 (43.4%)	MIS: 333 (67%) Elective: 486 (97%)	Not analysable
Croasdale 2022 [3] USA	None	Retrospective observational open label (before/after study)	CD: 91 (100%)	RHC/IC resection: 91 (100%)	MIS: 91 (100%) Conversions: 5 (5%) Elective: 91 (100%)	LOS: MD −1.21 (−2.21 to −0.21) days Readmissions: OR 1.78 (0.43 to 7.36) Reoperations: OR 0.22 (0.02 to 2.25) Complications: OR 1.07 (0.45 to 2.58)
D'Andrea 2019 [23] USA	None	Retrospective observational open label (before/after study)	Not specified, *n* = 460	CP: 49 (11%) Partial colectomy: 179 (39%) TAC: 66 (14%) TPC: 36 (8%) SBR: 66 (14%) Stoma reversal: 64 (14%)	MIS: 316 (67%) Elective: 460 (100%)	LOS: MD −1.10 (−2.08 to −0.12) days Readmissions: OR 0.74 (0.41 to 1.36) Reoperations: OR 0.75 (0.34 to 2.30) Complications: OR 0.33 (0.22 to 0.49)
Ding 2025 [24] China	Government and non‐profit funding	Retrospective observational open label (before/after study)	UC: 216 (100%)	TPC + IPAA: 216 (100%)	MIS: 156 (72%) Elective: 196 (91%)	LOS: MD −4.83(−7.73 to −1.94) days Complications: OR 0.32 (0.14 to 0.72)
Fabrizio 2017 [25] USA	Government funding	Retrospective observational open label (before/after study)	Not specified, *n* = 89	Not specified for IBD cohort	Not specified for IBD cohort	Readmissions: OR 0.87 (0.42 to 1.81)
Haering 2019 [21] USA (abstract only)	Not reported	Retrospective observational open label (before/after study)	CD: 151 (100%)	Not reported	Not reported	LOS: MD −1.00 (−1.80 to −0.20) days
Kariv 2007 [26] USA	Not reported	Retrospective observational open label, parallel group with matched controls	UC or IDC: 186 (96%) FAP: 8 (4%)	CP + IPAA: 66 (34%) TPC + IPAA: 128 (66%) Diverting ileostomy: 188 (97%)	MIS: 0 Elective: 186 (100%)	LOS: MD −0.70 (−1.78 to 0.38) days Readmissions: OR 1.28 (0.64 to 2.53) Complications: OR 1.11 (0.59 to 2.06)
Kelm 2024 [27] Germany	Unfunded. Lead author is the executive director of an ERAS company	Prospective observational open label (before/after study)	CD: 83 (100%)	IC resection: 83 (100%)	MIS: 69 (83%) Elective: not specified	LOS: MD −5.50 (−7.90 to −3.10) days Readmissions: OR 0.13 (0.02 to 1.03) Complications: OR 0.10 (0.03 to 0.33)
Lendzion 2024 [28] Australia	None	Retrospective observational, open label, parallel group	CD: 74 (62%) UC: 45 (38%)	CP + ileostomy: 3 (3%) CP + IPAA: 14 (12%) EC fistula resection: 4 (3%) IC/RHC: 46 LHC: 1 (1%) Pouch excision: 1 (1%) Sigmoid/LAR: 7 (6%) TAC: 20 (17%) TPC + ileostomy: 8 (7%) TPC + IPAA: 14 (12%) SBR: 13 (11%) Ostomy creation: 64 (54)	MIS: 61 (51%) Conversion: 4 (3%) Elective: 96 (81%)	LOS: MD −2.75 (−4.52 to −0.98) days Readmissions: OR 0.67 (0.21 to 2.07) Reoperations: OR 0.17 (0.01 to 4.32) Complications: OR 0.62 (0.29 to 1.34)
Lin 2024 [29] Denmark	Government funding	Retrospective observational open label (before/after study)	CD: 252 (64%) UC: 138 (35%) IDC: 4 (1%)	IC/RHC: 126 (32%) Left‐sided resection: 6 (2%) Ostomy creation only: 7 (2%) Ostomy takedown: 36 (9%) Ostomy revision: 22 (6%) Proctectomy: 23 (6%) SBR: 9 (2%) STC: 143 (36%) TPC: 4 (1%) Other: 8 (2%) Ostomy creation: 235 (60%)	MIS: 346 (88%) Conversion: 32 (8%) Local: 9 (2%) Elective: 271 (69%)	LOS: MD −1.33 (−1.94 to −0.73) days Readmissions: OR 1.52 (0.89 to 2.60) Reoperations: OR 0.67 (0.37 to 1.23) Complications: OR 1.03 (0.66 to 1.60)
Liska 2020 [30] USA	Not reported	Retrospective observational open label (before/after study)	CD: 345 (51%) UC: 326 (49%)	IPAA: 142 (21%) Ostomy surgery only: 43 (6%) Proctectomy: 54 (8%) Segmental colectomy: 170 (25%) TAC + ileostomy: 202 (30%)	MIS: 351 (52%) Elective: 559 (83%)	LOS: MD −1.83 (−2.40 to −1.27) days Readmissions: OR 0.63 (0.38 to 1.02) Reoperations: OR 0.17 (0.02 to 1.33) Complications: OR 1.10 (0.74 to 1.39)
Mineccia 2020 [31] Italy	Not reported	Retrospective observational open label (before‐and‐after study) with matched controls	CD: 94 (100%)	IC resection: 94 (100%)	MIS: 76 (80%) Elective: Not reported	LOS: MD −1.83 (−2.81 to −0.86) days Readmissions: OR 1.00 (0.13 to 7.41) Complications: OR 0.49 (0.04 to 5.59)
Shah 2017 [32] USA	Government funding	Retrospective observational open label (before and after study)	Not specified, *n* = 121	Not specified for IBD cohort	Not specified for IBD cohort	Readmissions: OR 0.37 (0.14 to 0.95)
Smith 2023 [33] USA	Government funding	Retrospective observational open label (before‐and‐after study)	CD: 286 (61%) UC: 180 (39%)	APR/LAR/Hartman: 111 (24%) Partial colectomy: 221 (47%) Stoma reversal: 8 (2%) TAC/TPC: 74 (16%) Other: 52 (11%)	MIS: Not reported Elective: 466 (100%)	LOS: MD −1.17 (−1.95 to −0.39) days Readmissions: OR 1.25 (0.74 to 2.11) Complications: OR 0.80 (0.53 to 1.19)
Spinelli 2013 [34] Italy	Not reported	Retrospective observational open label (before‐and‐after study) with matched controls	CD: 90 (100%)	IC resection: 90 (100%) Ostomy creation: 0	MIS: 90 (100%) Conversion: 0 Elective: 90 (100%)	LOS: MD −1.50 (−2.51 to −0.49) days Readmissions: OR 0.67 (0.03 to 14.49) Complications: OR 1.04 (0.33 to 3.28)
Violante 2024 [36] USA	None	Retrospective observational open label (before‐and‐after study)	CD: 1 (0.5%) UC: 192 (95%) FAP: 7 (4.5%)	TPC + IPAA: 106 (53%) CP + IPAA: 94 (47%)	MIS: 200 (100%) Conversion: 12 (6%) Elective: 200 (100%)	LOS: MD −1.40 (−2.09 to −0.71) days Readmissions: OR 0.16 (0.05 to 0.48) Reoperations: OR 1.35 (0.29 to 6.18) Complications: OR 0.28 (0.13 to 0.58)
Zhu 2018 [35] China	Government funding	RCT, open label	CD: 32 (100%)	IC resection: 32 (100%) Ostomy creation: 0	MIS: 32 (100%) Conversion: 0 Elective: 32 (100%)	LOS: MD −4.76 (−6.61 to −2.91) days Readmissions: OR 3.19 (0.12 to 84.43) Reoperations: Not estimable Complications: OR 1.00 (0.12 to 8.13)

Abbreviations: CD, Crohn's disease; CI, Confidence interval; CP, Completion proctectomy; EC, Enterocutaneous; ERAS, Enhanced recovery after surgery; FAP, Familial adenomatous polyposis; ID, Identification; IDC, Indeterminate colitis; IPAA, Ileal pouch anal anastomosis; IC, ileocolic; LAR, Low anterior resection; LHC, Left hemicolectomy; LOS, Length of stay; MD, Mean difference; MIS, Minimally invasive surgery; OR, Odds ratio; RCT, Randomised controlled trial; RHC, Right hemicolectomy; SBR, Small bowel resection; STC, Subtotal colectomy; TAC, Total abdominal colectomy; TPC, Total proctocolectomy; UC, Ulcerative colitis.

Seventeen studies with 4524 patients were assessed in the pooled analysis [3, 21, 22, 23, 24, 25, 26, 27, 28, 29, 30, 31, 32, 33, 34, 35]. One additional study was included in the narrative synthesis only, as the comparator group sample size was not available, and study authors could not be reached [20]. Studies were published from 2007 to 2025, including 2199 patients treated with ERAS and 2325 with conventional care. Studies were conducted in the USA (*n* = 11), Australia (*n* = 1), China (*n* = 2), Denmark (*n* = 1), Germany (*n* = 1) and Italy (*n* = 2).

Studies included similar proportions of both men and women (range: 29%–68% males). Mean age ranged from 34 to 49 years. Sample sizes ranged from 32 to 963 participants. Patients from a variety of health statuses were represented, with most studies including many ASA III and ASA IV individuals [3, 23, 28, 29, 30, 31, 33, 34]. Nine studies did not report ASA status for their IBD cohort [20, 21, 22, 24, 25, 26, 27, 32, 36]. Eight studies reported on a mixed population of UC and CD [22, 23, 25, 28, 29, 30, 32, 33]. Four studies included UC patients alone [20, 24, 26, 36], and six studies included CD only [3, 21, 27, 31, 34, 35].

A range of major colorectal operations was included; however, some studies focused on a single operation. Four studies included only ileocolic resections [27, 31, 34, 35], and three included only proctectomies with ileal pouch‐anal anastomoses (IPAA) [24, 26, 36]. One study included open surgery only [26]. Three studies examined only minimally invasive surgery [3, 34, 35]. The remaining studies had laparoscopic surgery rates from 51% to 100%, although in three studies laparoscopic surgery rates were not reported for the IBD cohort [21, 25, 32]. Three studies had a substantial portion of emergency surgery patients included [28, 29, 30]. The remaining studies included almost entirely elective surgeries, or the proportion of emergency surgeries was not reported.

There was one RCT [35], and 17 observational studies: one prospective cohort pre‐ and post‐implementation study [27], two parallel group retrospective studies [26, 28], and 14 retrospective pre‐ and post‐implementation studies [3, 20, 21, 22, 23, 24, 25, 29, 30, 31, 32, 33, 34, 36]. For the pre‐ and post‐implementation studies, the ERAS intervention occurred at the hospital level. For parallel‐group studies, ERAS implementation occurred at the individual surgeon level [26, 28]. All studies were unblinded. Study duration ranged from 1 to 15 years. No studies were explicitly industry‐sponsored, although one study's lead author was executive director of a company marketing ERAS program implementation [27]. Six studies did not report on conflicts of interest or financial disclosures [20, 21, 26, 30, 31, 34].

### Risk of bias

Risk of bias assessments for each primary outcome are displayed visually in Supporting Information: Figures 1.1–1.4. One RCT was included in the analysis and was assessed at low risk of bias for all outcomes except for LOS, which was assessed as some concerns due to lack of blinding [35]. For non‐randomised studies, bias arose predominantly from the ‘confounding’ domain, due to lack of statistical analysis to account for a non‐randomised population. Only one study used propensity score matching for the most important variables to account for a non‐randomised sample [31].

### Hospital LOS

Sixteen studies reported LOS [3, 20, 21, 22, 23, 24, 26, 27, 28, 29, 30, 31, 33, 34, 35, 36], and 15 were included in the meta‐analysis. The difference in LOS across studies was shorter with ERAS (6.83 days non‐ERAS vs. 5.21 days ERAS; MD −1.62 days, 95% CI −2.03, −1.21, 15 studies, 4225 participants, *I* ^2^ = 66%, moderate certainty evidence; Figure 2A). Subgroup analysis demonstrated a significantly greater reduction in LOS in studies reporting high protocol fidelity (MD −2.52 days, 95% CI −3.36, −1.68) compared with studies in which protocol fidelity was not reported (MD −1.13 days, 95% CI −1.39 to −0.87, Supporting Information: Figure 2.1). There were no other subgroup differences for UC vs. CD; new ostomy vs. no ostomy; surgery types; or NSAIDs vs. no NSAIDs (Supporting Information: Figures 2.2–2.7). The funnel plot showed significant asymmetry indicating possible nonreporting bias (Supporting Information: Figure 2.8). Nonreporting bias was investigated in sensitivity analysis by excluding three smaller studies with wide confidence intervals on post‐hoc sensitivity analysis, which minimised heterogeneity (*I* ^2^ = 1%) while preserving the magnitude and statistical significance of results (Supporting Information: Figures 2.9–2.10). There were no other differences on sensitivity analysis (Supporting Information: Figures 2.11–2.15). Evidence certainty for this outcome was limited by serious risk of bias (Supporting Information: Table 1).

**FIGURE 2:**
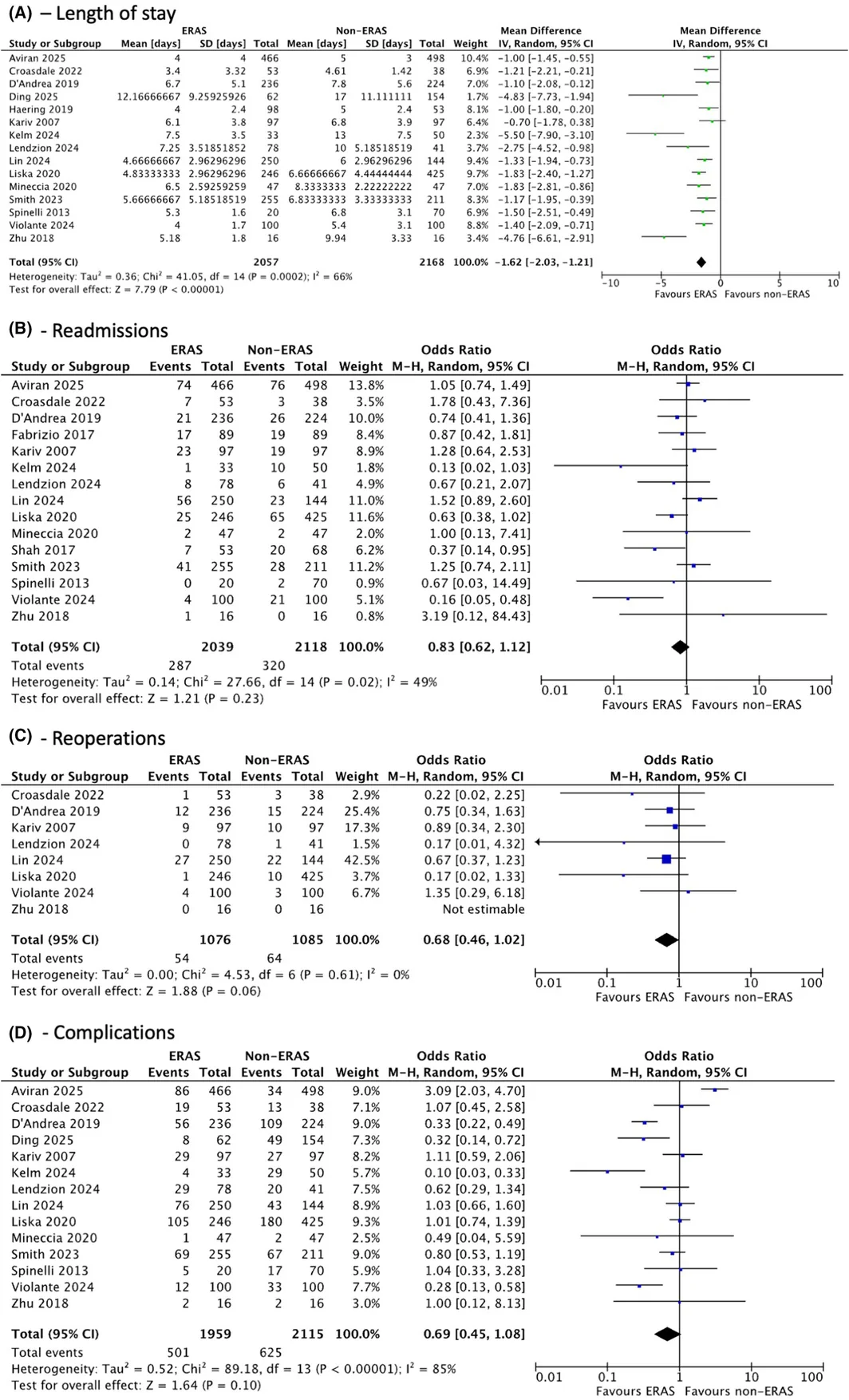
Forest plots for patients treated according to an enhanced recovery after surgery (ERAS) protocol versus non‐ERAS protocol for outcomes: (A) length of hospital stay (mean difference, days), (B) 30‐day readmissions, (C) 30‐day reoperations and (D) 30‐day complications.

### Readmissions

Sixteen studies reported hospital readmissions, and 15 were included in the meta‐analysis. Fourteen studies reported 30‐day readmissions [3, 20, 22, 23, 25, 26, 27, 28, 29, 32, 33, 34, 35, 36], one study reported 90‐day readmissions [31] and one study reported no time interval [30]. There was no difference in readmission rate across studies for ERAS compared to non‐ERAS (OR: 0.83, 95% CI 0.62, 1.12, 15 studies, 4157 participants, *I* ^2^ = 49%, low certainty evidence; Figure 2B). In subgroup analysis, studies reporting high protocol fidelity demonstrated a significant reduction in 30‐day readmissions with ERAS (OR 0.66, 95% CI 0.47–0.93; *I* ^2^ = 0%), whereas studies that did not report fidelity rates showed no significant association (OR 0.91, 95% CI 0.61–1.36; *I* ^2^ = 64%; Supporting Information: Figure 3.1). There were no other differences in subgroup and sensitivity analyses, including exclusion of studies reporting readmission intervals other than 30 days (Supporting Information: Figures 3.2–3.14). The funnel plot for this outcome was symmetric, with no obvious evidence of publication bias (Supporting Information: Figure 3.15). Evidence certainty was limited by serious risk of bias and serious imprecision (Supporting Information: Table 1).

### Reoperations

Nine studies reported reoperations, seven within 30 days [20, 23, 26, 28, 29, 35, 36], and two without a time interval [3, 30]. There was no difference in reoperations across studies for the ERAS group compared to non‐ERAS (OR: 0.68, 95% CI 0.46, 1.02, 8 studies, 2161 participants, *I* ^2^ = 0%, very low certainty evidence; Figure 2C). There were no differences in the planned subgroup analysis (Supporting Information: Figures 4.1–4.6). Sensitivity analyses with a fixed‐effects model or when analysed according to risk difference demonstrated reduced 30‐day reoperations in the ERAS group (Supporting Information: Figures 4.7–4.8). There were no other differences on sensitivity analysis (Supporting Information: Figures 4.9–4.11). Funnel plot was not assessed, as recommended for outcomes with fewer than 10 studies [17]. Evidence certainty for this outcome was limited by very serious risk of bias and serious imprecision (Supporting Information: Table 1).

### Complications

Fifteen studies reported postoperative complications [3, 20, 22, 23, 24, 26, 27, 28, 29, 30, 31, 33, 34, 35, 36], and 14 were included in the meta‐analysis. There was no difference in total 30‐day complications across studies for ERAS compared to non‐ERAS (OR: 0.69, 95% CI 0.45, 1.08, 14 studies, 4074 participants, *I* ^2^ = 85%, low certainty evidence; Figure 2D). In subgroup analysis, high‐fidelity ERAS studies had a greater reduction in complications than studies where protocol fidelity was not recorded (Supporting Information: Figure 5.1). Similarly, ERAS protocols in studies without NSAID use had fewer complications compared to studies where NSAIDs were used (Supporting Information: Figure 5.2). ERAS protocols designed for a single surgery type (ileocolic resection in CD or IPAA in UC) also had reduced complications compared to those designed for multiple types of procedures (Supporting Information: Figure 5.3). There were no other subgroup differences (Supporting Information: Figures 5.4–5.6). In the sensitivity analysis, a fixed‐effects model showed reduced complications with ERAS (Supporting Information: Figure 5.7), as did exclusion of the unpublished Aviran et al. study, which was a clear visual outlier in the primary forest plot (Supporting Information: Figure 5.8). Heterogeneity was not reduced in other sensitivity analysis (Supporting Information: Figures 5.7–5.13). The funnel plot for this outcome was symmetric (Supporting Information: Figure 5.13). Evidence certainty for this outcome was limited by serious risk of bias and serious imprecision (Supporting Information: Table 1).

With respect to individual complications, 8 studies reported on deaths [3, 23, 26, 29, 30, 31, 34, 35], 11 reported on anastomotic leaks [3, 20, 23, 24, 26, 27, 28, 29, 30, 34, 36], 5 reported on UTI [3, 23, 30, 33, 36], 11 reported on bleeding [20, 22, 23, 24, 26, 28, 30, 31, 33, 34, 36], 8 reported on postoperative ileus [3, 23, 24, 27, 30, 34, 35, 36] and 11 reported on SSIs [3, 23, 24, 26, 27, 28, 30, 33, 34, 35, 36]. There was no difference between groups in mortality (Supporting Information: Figure 6.1), anastomotic leak (Supporting Information: Figure 6.2), UTI (Supporting Information: Figure 6.3), bleeding (Supporting Information: Figure 6.4), ileus (Supporting Information: Figure 6.5) or venous thromboembolic complications (Supporting Information: Figure 6.6). Heterogeneity was minimal (*I* ^2^ < 25%) for mortality, leaks, UTI and venous thromboembolic complications, but was moderate for bleeding (*I* ^2^ = 60%) and postoperative ileus (*I* ^2^ = 67%). Funnel plots for postoperative bleeds and anastomotic leaks were symmetrical (Supporting Information: Figures 6.7 and 6.8). Funnel plots were not generated for other complications, as they had fewer than 10 included studies each.

SSIs were reduced in the ERAS group compared to non‐ERAS (OR: 0.53, 95% CI 0.31, 0.91, 11 studies, 2622 participants, *I* ^2^ = 65%, Supporting Information: Figure 6.9). The funnel plot demonstrated no asymmetry (Supporting Information: Figure 6.10). When SSIs were subclassified according to superficial, deep, and organ/space infections, there was no difference between groups (Supporting Information: Figures 6.11–6.14).

### ERAS components

All 24 items recommended by the 2018 ERAS society guidelines for colorectal surgery were studied, although no single study included every aspect recommended [1]. ERAS items were variably implemented in each study (Table 2). The most reported ERAS components were MIS approach (*n* = 16), early mobilisation (*n* = 14) and early feeding (*n* = 14). Only one study reported postoperative hyperglycaemia prevention [23]. Compliance with individual ERAS protocol components ranged from 10%–100% and was reported on in only eight out of 18 studies.

**TABLE 2:** Adherence to enhanced recovery after surgery (ERAS) program components for patients with inflammatory bowel disease.

ERAS component	Aviran 2025	Chang 2017	Croasdale 2022	D'Andrea 2020	Ding 2025	Fabrizio 2017	Haering 2019	Kariv 2007	Kelm 2024	Lendzion 2024	Lin 2024	Liska 2020	Mineccia 2020	Shah 2017	Smith 2023	Spinelli 2013	Violante 2024	Zhu 2018
Preoperative patient education	RNR	NI	NI	38%	NI	NI	NI	RNR	90%^f^	NI	RNR	RNR	NI	RNR	RNR	RNR	NI	RNR
Preoperative optimisation	NI	NI	NI	77%^a^	100%^e^	NI	NI	NI	90%^f^	NI	RNR	RNR	NI	NI	NI	NI	NI	NI
Prehabilitation	NI	NI	NI	NI	NI	NI	NI	NI	90%^f^	NI	NI	NI	NI	NI	NI	NI	NI	NI
Nutrition assessment	NI	NI	NI	NI	100%^e^	NI	NI	NI	90%^f^	NI	RNR	RNR	NI	NI	NI	NI	NI	NI
Anaemia screening	NI	NI	NI	NI	100%^e^	NI	NI	NI	90%^f^	NI	RNR	RNR	NI	NI	NI	NI	NI	NI
Prevention of nausea and vomiting	RNR	NI	RNR	57%^b^	NI	RNR	NI	NI	90%^f^	NI	RNR	RNR	NI	NI	NI	NI	NI	NI
Selective premedication	NI	NI	NI	NI	NI	RNR	NI	NI	90%^f^	NI	RNR	RNR	~95%^i^	NI	NI	RNR	NI	NI
Prophylactic intravenous antibiotics	NI	NI	NI	100%	NI	NI	NI	NI	90%^f^	NI	RNR	RNR	NI	RNR	NI	NI	NI	NI
Appropriate bowel preparation	NI	NI	NI	64%^c^	NI	NI	NI	NI	90%^f^	NI	RNR	RNR	~95%^i^	RNR	RNR	100%	NI	100%
Preoperative euvolaemia	NI	NI	NI	NI	NI	NI	NI	NI	90%^f^	NI	RNR	RNR	~95%^i^	RNR	NI	100%	NI	100%
No fasting and carbohydrate drink	NI	NI	RNR	38%	NI	RNR	NI	NI	90%^f^	RNR	RNR	RNR	~95%^i^	RNR	NI	100%	NI	100%
Standard anaesthetic protocol	RNR	NI	NI	NI	100%^e^	RNR	NI	NI	83%^f^	NI	RNR	NI	NI	RNR	NI	RNR	NI	NI
Intraoperative normovolaemia	NI	NI	NI	NI	NI	NI	NI	NI	83%^f^	NI	RNR	RNR	~95%^i^	RNR	NI	100%	NI	100%
Normothermia	NI	NI	RNR	NI	NI	RNR	NI	NI	83%^f^	NI	RNR	RNR	NI	NI	RNR	NI	NI	100%
MIS approach	62%	67%	100%	73%	100%^e^	RNR	RNR	NI	91%	46%	90%	61%	79%	RNR	RNR	100%	NI	100%
No intra‐abdominal drains	NI	NI	NI	NI	NI	NI	NI	NI	83%^f^	NI	RNR	NI	NI	NI	NI	10%	NI	88%
No nasogastric tube	RNR	NI	NI	NI	NI	NI	NI	RNR	75%^f^	72%^g^	RNR	84%	~95%^i^	NI	NI	100%	NI	100%
Multimodal analgesia	RNR	NI	RNR	23–78%	NI	RNR	NI	RNR	75%^f^	NI	RNR	81–99%	~95%^i^	RNR	RNR	85%	NI	RNR
Thromboprophylaxis	RNR	NI	NI	NI	100%^e^	NI	NI	NI	75%^f^	NI	RNR	RNR	NI	RNR	RNR	NI	NI	NI
Postoperative normovolaemia	NI	NI	RNR	16%	NI	NI	NI	NI	75%^f^	NI	RNR	RNR	NI	RNR	NI	100%	NI	100%
Urinary catheter removal 1–3 days	RNR	NI	RNR	NI	100%^e^	NI	NI	NI	75%^f^	72%^g^	RNR	97%h	~95%^i^	NI	RNR	100%^h^	NI	100%h
Prevent hyperglycemia	NI	NI	NI	RNR	NI	NI	NI	NI	NI	NI	NI	NI	NI	NI	NI	NI	NI	NI
Postoperative early feeding	RNR	NI	RNR	82%^d^	100%^e^	NI	NI	RNR	75%^f^	72%^g^	RNR	76%	~95%^i^	RNR	RNR	80%	NI	88%
Early mobilisation	RNR	NI	RNR	65–77%	100%^e^	RNR	NI	RNR	75%^f^	72%^g^	RNR	RNR	94%	NI	RNR	90%	NI	94%

*Note*: Categories derived from Guidelines for Perioperative Care in Elective Colorectal Surgery: Enhanced Recovery After Surgery (ERAS®) Society Recommendations: 2018.

Abbreviations: NI, Not included (in written description of ERAS protocol); RNR, Rate not reported (but was included in study's ERAS protocol description).

^a^ Diabetic testing only.

^b^ Reported as compliance with all pre‐medication.

^c^ Oral antibiotic rate only, mechanical bowel preparation was optional and the rate was not reported.

^d^ Clear liquid diet on post‐operative day 1.

^e^ ERAS group was defined as patients who adhered to all perioperative ERAS components.

^f^ Preoperative, intraoperative, and post‐operative ERAS components were each assessed as a package.

^g^ Compliance with ERAS items was measured as the ERAS whole package, which was assigned in the post‐operative orders.

^h^ Catheter removal on POD1.

^i^ Compliance with the entire ERAS protocol except where noted.

Multimodal analgesia was described in 13 studies. Perioperative NSAIDs were included in seven studies [22, 23, 25, 30, 32, 34, 35], selective neuraxial anaesthesia in six [23, 25, 29, 30, 31, 32] and regional nerve blocks (e.g., transversus abdominal block) in five [22, 23, 25, 30, 33]. Most studies reported multimodal analgesia from the preoperative to the postoperative periods; however, only one study reported the discharge prescription [22].

Primary outcomes were investigated in a sensitivity analysis according to ERAS components. The largest reduction in LOS was in studies with protocols including preoperative nutrition assessment, anaemia screening, preoperative euvolemia, preoperative optimisation (preoperative risk assessment for comorbidities, smoking and alcohol cessation), intraoperative normovolemia, intraoperative normothermia and avoidance of intra‐abdominal drains. These all led to >2 day decrease in LOS compared to 1.62 days for all pooled protocols (Supporting Information: Table 2). No individual ERAS protocol components were associated with reduced readmissions (Supporting Information: Table 3). ERAS protocols including preoperative nausea and vomiting prevention, avoidance of preoperative fasting, MIS approach, postoperative normothermia, early urinary catheter removal, early postoperative feeding and mobilisation were each associated with statistically significant decreased odds of reoperation compared to non‐ERAS (Supporting Information: Table 4). Finally, protocols including preoperative optimisation, prehabilitation and hyperglycemia prevention (although the latter two were limited to single small studies) were all associated with statistically significant decreased odds of complications (Supporting Information: Table 5).

## DISCUSSION

The results of this systematic review and meta‐analysis show that in adult patients undergoing colorectal surgery for IBD, ERAS was associated with a reduced mean LOS by 1.62 days with no difference in 30‐day readmissions, reoperations, or complications. This LOS reduction is smaller than that reported for colorectal surgery for cancer. A 2011 Cochrane review reported an LOS reduction of 2.94 days with ERAS [2]. One possible explanation for this perceived reduced ERAS effectiveness in IBD compared to cancer is temporal variation in practice during the 15 years between these two meta‐analyses. However, it may also be that ERAS is less effective in patients with IBD due to disease‐specific differences such as inflamed tissues, more nutritional compromise and immunosuppression.

Studies reporting high ERAS protocol fidelity were associated with better LOS, readmissions and complications, with high‐compliance studies reporting better outcomes than those in which compliance was not reported. However, these findings should be interpreted cautiously. Studies reporting high protocol fidelity may differ systematically from studies with low or unreported fidelity in ways unrelated to ERAS compliance itself, including centre experience, implementation of multidisciplinary perioperative care, reporting quality and other unmeasured aspects of perioperative practice. Consequently, the observed associations cannot be attributed solely to protocol fidelity and should be considered hypothesis‐generating. In addition to ERAS compliance, considerations for how to tailor ERAS protocols for patients with IBD may help to improve outcomes [37]. Our findings generate the hypothesis that procedure‐specific ERAS pathways with greater emphasis on preoperative optimisation may offer additional benefit for patients with IBD and should be evaluated in future randomised trials. Our subgroup analysis suggested greater treatment effects in studies restricted to a single operative procedure (e.g., ileocolic resection or IPAA) than in studies including multiple colorectal procedures. This may reflect greater procedure‐specific tailoring of ERAS pathways; however, most studies did not describe their protocols in sufficient detail to confirm this, and greater homogeneity of the study populations may also have contributed to the observed differences. In our sensitivity analysis, many of the pre‐operative phase ERAS components led to reductions in LOS; however, these components were more variable and had poorer uptake than for peri‐operative and postoperative components. Therefore, increased focus on the pre‐operative phase may be an area of opportunity to improve LOS in patients with IBD. However, these subgroup and sensitivity analyses were based on study‐level comparisons and should therefore be considered hypothesis‐generating. Future adequately powered randomised trials are needed to evaluate procedure‐specific IBD ERAS pathways and determine whether enhanced preoperative optimisation provides additional benefit beyond standard colorectal ERAS protocols.

NSAIDS continue to be a controversial ERAS element in IBD due to concerns about increased risk of anastomotic leak and early CD recurrence [1, 38, 39]. While our study did not show a significant difference for LOS, readmissions or re‐operations, it did show fewer complications in the ERAS groups that did not use NSAIDS, driven by a trend towards less postoperative bleeding with no difference in anastomotic leaks.

Our meta‐analysis builds upon other contemporary reviews [37, 40]. Peng et al. [41] found similar reductions in LOS and no effect on readmissions or complications but included three studies outside our criteria: one comparing IBD to non‐IBD surgery and two paediatric studies [42, 43]. Vigorita et al. [37] performed a systematic review without meta‐analysis, including many uncontrolled studies excluded here. As in our findings, they noted limited reporting of preoperative optimisation and an emphasis on immediate preoperative and postoperative interventions. Our review adds 13 more studies to the prior meta‐analysis, and a formal evidence‐certainty assessment to allow clinicians and researchers to make informed decisions, which has not been performed previously on this topic. We also identified a dose–response effect according to ERAS‐protocol fidelity, and identified individual ERAS items associated with maximal effectiveness, providing additional supporting evidence for an IBD‐specific ERAS protocol.

Future research should focus on adequately powered randomised controlled trials evaluating ERAS pathways specifically designed for patients with IBD. Rather than comparing ERAS with conventional care alone, these studies should evaluate procedure‐specific ERAS pathways that incorporate enhanced preoperative optimisation, including nutritional assessment, anaemia management and prehabilitation where appropriate. Standardised reporting of protocol adherence and individual ERAS components will also be essential to identify which elements contribute most to improved outcomes.

## LIMITATIONS

One limitation is that the pooled analyses combined data from one small randomised controlled trial and predominantly observational studies. We elected to pool these data because only a single RCT involving 32 patients was available [35] and separate randomised evidence alone would have been insufficient to provide meaningful pooled estimates for any outcome. Pooling randomised and observational evidence allowed incorporation of the strongest available evidence while improving precision. However, observational studies remain susceptible to residual confounding and secular changes in perioperative care, particularly because most used before‐and‐after designs. To assess whether inclusion of the RCT influenced our findings, we performed a sensitivity analysis excluding the randomised trial, which did not materially alter the pooled estimates or overall conclusions. Evidence certainty was very low to moderate according to our GRADE assessment. There was also moderate to high statistical heterogeneity for most primary outcomes. Included trials remained heterogenous in ways that could not be separated into subgroups. For example, all trials included different perioperative treatment protocols, and many did not describe their ERAS protocol in detail or report compliance. Most studies also did not define care in the non‐ERAS arm. The study by Aviran et al. was a notable outlier in the complications meta‐analysis and contributed substantially to the observed heterogeneity. Sensitivity analysis excluding this study demonstrated a statistically significant reduction in postoperative complications with ERAS. However, because the study met our predefined eligibility criteria, there was no methodological justification for its exclusion from the primary analysis, which therefore included all eligible studies. While our subgroup and sensitivity analyses minimised detectable statistical heterogeneity, unmeasured factors may still have influenced results.

## CONCLUSIONS

In this meta‐analysis, ERAS for adult patients with IBD was shown to reduce length of stay with no difference in 30‐day readmissions, reoperations, or complications. Our findings generate the hypothesis that procedure‐specific ERAS pathways with greater emphasis on preoperative optimisation may offer additional benefit for patients with IBD and should be evaluated in future randomised trials.

## AUTHOR CONTRIBUTIONS

**Garrett Johnson:** Methodology; writing – review and editing; writing – original draft; investigation; validation; visualization; software; formal analysis; data curation. **Evangelia Theodosopoulos:** Conceptualization; methodology; writing – original draft; writing – review and editing; formal analysis; data curation. **Jordyn Vernon:** Writing – review and editing; formal analysis; data curation. **Selina Schmocker:** Writing – review and editing; project administration; data curation; investigation. **Hilalion (San) Ahn:** Investigation; formal analysis; writing – review and editing; data curation. **Javier Gomez:** Writing – review and editing; data curation; formal analysis; investigation. **Mantaj S. Brar:** Writing – review and editing; methodology; validation; supervision; investigation. **Aman Pooni:** Supervision; writing – review and editing; investigation. **Erin D. Kennedy:** Conceptualization; investigation; writing – original draft; writing – review and editing; methodology; project administration; supervision.

## CONFLICT OF INTEREST STATEMENT

The authors declare no conflicts of interest.

## ETHICS STATEMENT

Ethical approval was not required for this systematic review and meta‐analysis, as the study used data from previously published studies and did not involve the collection of new individual‐level patient data.

## Supporting information


**Data S1.** PRISMA 2020 Checklist.

**Data S2.** PRISMA 2020 Checklist.

**Supporting Information S1.** Search strategy and search log.

**Supporting Information S2.** Reasons for excluded studies.

**Supporting Information Table S3.** Detailed Characteristics of Ongoing studies.

**Supporting Information S4.** Supplementary tables and figures for risk of bias assessments, Grade assessment for primary outcomes, subgroup analyses, sensitivity analyses and funnel plots.

## Data Availability

The data that supports the findings of this study are available in the supplementary material of this article.

## References

[1] Gustafsson UO , Scott MJ , Hubner M , Nygren J , Demartines N , Francis N , et al. Guidelines for perioperative Care in Elective Colorectal Surgery: enhanced recovery after surgery (ERAS®) society recommendations: 2018. World J Surg. 2019;43:659–695. 10.1007/s00268-018-4844-y 30426190

[2] Spanjersberg WR , Reurings J , Keus F , van Laarhoven CJHM , Cochrane Colorectal Cancer Group . Fast track surgery versus conventional recovery strategies for colorectal surgery. Cochrane Database Syst Rev. 2011;2011:CD007635. 10.1002/14651858.CD007635.pub2 PMC13061361 21328298

[3] Croasdale DR , Su EM , Olutola OE , Polito CP , Ata A , Keenan M , et al. The effect of an enhanced recovery program on elective right hemicolectomies for Crohn's disease vs. colon cancer: a retrospective cohort analysis. Am Surg. 2022;88:120–125. 10.1177/0003134820982573 33356439

[4] Casanova MJ , Chaparro M , Molina B , Merino O , Batanero R , Dueñas‐Sadornil C , et al. Prevalence of malnutrition and nutritional characteristics of patients with inflammatory bowel disease. J Crohns Colitis. 2017;11:1430–1439. 10.1093/ecco-jcc/jjx102 28981652

[5] Avellaneda N , Pellino G , Maroli A , Tottrup A , Bislenghi G , Colpaert J , et al. Short‐term outcomes of surgical treatment for primary ileocaecal Crohn's disease: results of the Crohn's(urg) study, a multicentre, retrospective, comparative analysis between inflammatory and complicated phenotypes. Color Dis. 2024;26:1415–1427. 10.1111/codi.17056 38858815

[6] Avellaneda N , Maroli A , Pellino G , Carvello M , Tottrup A , Bislenghi G , et al. Long‐term comparative outcomes after ileocecal resection for inflammatory versus complicated Crohn's disease. A multicenter, retrospective study (Crohn's‐Urg). Dig Liver Dis. 2025;57(10):1920–1926. 10.1016/j.dld.2025.07.009 40796414

[7] Group EERC . An international assessment of the adoption of enhanced recovery after surgery (ERAS®) principles across colorectal units in 2019–2020. Color Dis. 2021;23:2980–2987. 10.1111/codi.15863 34365718

[8] Page MJ , McKenzie JE , Bossuyt PM , Bossuyt PM , Boutron I , Hoffmann TC , et al. The PRISMA 2020 statement: an updated guideline for reporting systematic reviews. Int J Surg. 2021;88:105906. 10.1016/j.ijsu.2021.105906 33789826

[9] Higgins JPT , Lasserson T , Thomas J , Flemyng E , Churchill R . Methodological Expectations of Cochrane Intervention Reviews. Cochrane Intervent Rev. 2023. https://www.cochrane.org/authors/handbooks‐and‐manuals/mecir‐manual

[10] Horan TC , Gaynes RP , Martone WJ , Jarvis WR , Emori TG . CDC definitions of nosocomial surgical site infections, 1992: a modification of CDC definitions of surgical wound infections. Infect Control Hosp Epidemiol. 1992;13:606–608. 1334988

[11] Higgins JPT , Savović J , Page MJ , Elbers R , Sterne J . Chapter 8: assessing risk of bias in a randomized trial. Cochrane handbook for systematic reviews of interventions. London: Cochrane; 2019. https://www.cochrane.org/authors/handbooks‐and‐manuals/handbook/current/chapter‐08

[12] Higgins JPT , Sterne J , Savović J , Page M , Hróbjartsson A , Boutron I , et al. A revised tool for assessing risk of bias in randomized trials. Cochrane Database Syst Rev. 2016;10(Suppl 1):29–31.

[13] Sterne J , Higgins J . ROBINS‐I V2 tool. 2024. https://sites.google.com/site/riskofbiastool/welcome/robins‐i‐v2. Accessed 19 August 2025.

[14] The GRADE Working Group . GRADE handbook for grading quality of evidence and strength of recommendations. 2013.

[15] Wan X , Wang W , Liu J , Tong T . Estimating the sample mean and standard deviation from the sample size, median, range and/or interquartile range. BMC Med Res Methodol. 2014;14:135. 10.1186/1471-2288-14-135 PMC4383202 25524443

[16] Higgins JPT , Deeks JJ . Chapter 6: choosing effect measures and computing estimates of effect. Cochrane handbook for systematic reviews of interventions. London: Cochrane; 2023. https://www.cochrane.org/authors/handbooks‐and‐manuals/handbook/current/chapter‐06#section‐6‐5‐2‐8. Accessed 13 August 2025.

[17] Page MJ , Higgins JPT , Sterne J . Chapter 13: assessing risk of bias due to missing evidence in a meta‐analysis. Cochrane handbook for systematic reviews of interventions. London: Cochrane; 2024. https://www.cochrane.org/authors/handbooks‐and‐manuals/handbook/current/chapter‐13. Accessed 13 August 2025.

[18] Higgins JPT , Thompson SG , Deeks JJ , Altman DG . Measuring inconsistency in meta‐analyses. BMJ. 2003;327:557–560. 10.1136/bmj.327.7414.557 PMC192859 12958120

[19] NCT02997293 . Impact of the Enhanced Recovery After Surgery System for Colorectal Surgery. 2016. https://trialsearch.who.int/Trial2.aspx?TrialID=NCT02997293

[20] Chang M , Larson D , Dozois E , Mathis K , Kelley S , Lightner A . Enhanced recovery protocols in patients with ulcerative colitis. Dis Colon Rectum. 2017;60:e426EP–e427EP.

[21] Haering D , Ferrari L , Fichera A , Krane M . The role of enhanced recovery protocols in the perioperative care of patients with Crohn's disease. 2019;156:S1453.

[22] Aviran E , Assaf D , Zaghiyan KN , Fleshner P . Has the use of enhanced recovery protocols in colorectal surgery increased postoperative bleeding complications? Dis Colon Rectum. 2025;68:366EP–372EP. 10.1097/DCR.0000000000003581 39625405

[23] D'Andrea AP , Khetan P , Miller R , Sylla P , Divino CM . Outcomes after bowel resection for inflammatory bowel disease in the era of surgical care bundles and enhanced recovery. J Gastrointest Surg. 2020;24:123–131. 10.1007/s11605-019-04362-2 31468328

[24] Ding W , Dai Z , Cui L , Wu X , Zhou W , Ding Z , et al. Perioperative enhanced recovery program implementation improves clinical outcomes in patients with ulcerative colitis after total proctocolectomy with ileal pouch‐anal anastomosis. Int J Color Dis. 2025;40:38. 10.1007/s00384-025-04824-x PMC11825569 39945848

[25] Fabrizio AC , Grant MC , Siddiqui Z , Alimi Y , Gearhart SL , Wu C , et al. Is enhanced recovery enough for reducing 30‐d readmissions after surgery? J Surg Res. 2017;217:45–53. 10.1016/j.jss.2017.04.007 28602223

[26] Kariv Y , Delaney CP , Senagore AJ , Manilich EA , Hammel JP , Church JM , et al. Clinical outcomes and cost analysis of a ‘fast track’ postoperative care pathway for ileal pouch‐anal anastomosis: a case control study. Dis Colon Rectum. 2007;50:137–146. 10.1007/s10350-006-0760-6 17186427

[27] Kelm M , Wagner L , Widder A , Pistorius R , Wagner JC , Schlegel N , et al. Perioperative enhanced recovery concepts significantly improve postoperative outcome in patients with Crohn's disease. J Crohns Colitis. 2024;18:1857–1862. 10.1093/ecco-jcc/jjae090 38878058

[28] Lendzion RJ , Sidhu A , D'Souza B . Peri‐operative outcomes of patients with inflammatory bowel disease after the introduction of an ERAS protocol – a retrospective cohort review. Open Access Surgery. 2024;17:1–9.

[29] Lin V , Poulsen JK , Juvik AF , Roikjær O , Gögenur I , Fransgaard T . The implementation of an inflammatory bowel disease‐specific enhanced recovery after surgery protocol: an observational cohort study. Tech Coloproctol. 2024;28:58. 10.1007/s10151-024-02933-3 PMC11127850 38796600

[30] Liska D , Cengiz TB , Novello M , Aiello A , Stocchi L , Hull TL , et al. Do patients with inflammatory bowel disease benefit from an enhanced recovery pathway? Inflamm Bowel Dis. 2020;26:476–483. 10.1093/ibd/izz172 31372647

[31] Mineccia M , Menonna F , Germani P , Gentile V , Massucco P , Rocca R , et al. A retrospective study on efficacy of the ERAS protocol in patients undergoing surgery for Crohn disease: a propensity score analysis. Dig Liver Dis. 2020;52:625–629. 10.1016/j.dld.2020.01.006 32085992

[32] Shah PM , Johnston L , Sarosiek B , Harrigan A , Friel CM , Thiele RH , et al. Reducing readmissions while shortening length of stay: the positive impact of an enhanced recovery protocol in colorectal surgery. Dis Colon Rectum. 2017;60:219–227. 10.1097/DCR.0000000000000748 PMC5268399 28059919

[33] Smith BP , Jones BA , Cofer KD , Hollis RH , Shao C , Gleason L , et al. Racial disparities in postoperative outcomes persist for patients with inflammatory bowel disease under a colorectal enhanced recovery program. Am J Surg. 2023;226:227–232. 10.1016/j.amjsurg.2023.04.009 PMC10524897 37120415

[34] Spinelli A , Bazzi P , Sacchi M , Danese S , Fiorino G , Malesci A , et al. Short‐term outcomes of laparoscopy combined with enhanced recovery pathway after ileocecal resection for Crohn's disease: a case‐matched analysis. J Gastrointest Surg. 2013;17:126–132. 10.1007/s11605-012-2012-5 22948838

[35] Zhu Y , Xiang J , Liu W , Cao Q , Zhou W . Laparoscopy combined with enhanced recovery pathway in ileocecal resection for Crohn's disease: a randomized study. Gastroenterol Res Pract. 2018;2018:9648674. 10.1155/2018/9648674 PMC6252211 30534152

[36] Violante T , Ferrari D , Gomaa IA , Rumer KK , D'Angelo ALD , Behm KT , et al. Evolution of laparoscopic ileal pouch‐anal anastomosis: impact of enhanced recovery program, medication changes, and staged approaches on outcomes. J Gastrointest Surg. 2024;28:501–506. 10.1016/j.gassur.2024.01.005 38583902

[37] Vigorita V , Cano‐Valderrama O , Celentano V , Vinci D , Millán M , Spinelli A , et al. Inflammatory bowel diseases benefit from enhanced recovery after surgery [ERAS] protocol: a systematic review with practical implications. J Crohns Colitis. 2022;16:845–851. 10.1093/ecco-jcc/jjab209 34935916

[38] Moninuola OO , Milligan W , Lochhead P , Khalili H . Systematic review and meta‐analysis: association between acetaminophen and non‐steroidal anti‐inflammatory drugs (NSAIDs) and risk of Crohn's disease and ulcerative colitis exacerbation. Aliment Pharmacol Ther. 2018;47:1428–1439. 10.1111/apt.14606 PMC5992031 29620794

[39] Hayazei M , Abed M , Atıcı SD , Ahmad S , El‐Hussuna A . Systematic review of the effect of non‐steroidal anti‐inflammatory drugs on the exacerbation of inflammatory bowel disease. Turk J Colorectal Dis. 2023;33:92–102. 10.4274/tjcd.galenos.2023.2023-3-1

[40] Peng D , Cheng Y‐X , Tao W , Tang H , Ji GY . Effect of enhanced recovery after surgery on inflammatory bowel disease surgery: a meta‐analysis. World J Clin Cases. 2022;10:3426–3435. 10.12998/wjcc.v10.i11.3426 PMC9048538 35611189

[41] Meunier A , Sorce G , Hardy P‐Y , Coimbra C , Decker E , Joris J . Comparison of outcome after right colectomy with an enhanced recovery programme in patients with inflammatory bowel disease and patients operated on for other conditions: a monocentric retrospective study. Int J Color Dis. 2021;36:757–763. 10.1007/s00384-020-03830-5 33423143

[42] Vrecenak JD , Mattei P . Fast‐track management is safe and effective after bowel resection in children with Crohn's disease. J Pediatr Surg. 2014;49:99–103. 10.1016/j.jpedsurg.2013.09.038 24439590

[43] West MA , Horwood JF , Staves S , Jones C , Goulden MR , Minford J , et al. Potential benefits of fast‐track concepts in paediatric colorectal surgery. J Pediatr Surg. 2013;48:1924–1930. 10.1016/j.jpedsurg.2013.02.063 24074669

