# Supplementary material for: Enhanced recovery after colorectal surgery in patients with inflammatory bowel disease to reduce hospital length of stay: A systematic review and meta‐analysis
Source: Colorectal Dis. 2026 Sep 29;28(10):e70642. doi: 10.1111/codi.70642 (PMC13624278; doi:10.1111/codi.70642)
Supplement: Supplementary file 3 [file CODI-28-0-s002.docx]

**Enhanced recovery after colorectal surgery in patients with inflammatory bowel disease: Subgroup, sensitivity analysis, and funnel plots**

[1. Risk of bias assessments and summary of findings	1](#_Toc224215364)

[2. Length of Stay (LOS)	7](#_Toc224215365)

[3. 30-day Readmissions	14](#_Toc224215366)

[4. 30-day Reoperations	21](#_Toc224215367)

[5. Total complications	26](#_Toc224215368)

[6. Complication subcategories	37](#_Toc224215369)

# 1. Risk of bias assessments and summary of findings

Figure 1.1 Length of stay (mean difference, days), for enhanced recovery after surgery (ERAS) protocol vs non-ERAS A) Forest plot, and B) risk of bias assessments according to ROBINS-I tool for observational studies and C) ROB 2.0 tool for randomized controlled trials

Figure 1.2 30-day readmissions for enhanced recovery after surgery (ERAS) protocol vs non-ERAS A) Forest plot, and B) risk of bias assessments according to ROBINS-I tool for observational studies and C) ROB 2.0 tool for randomized controlled trials.

Figure 1.3 30-day reoperations after enhanced recovery after surgery (ERAS) protocol vs non-ERAS A) Forest plot, and B) risk of bias assessments according to ROBINS-I tool for observational studies and C) ROB 2.0 tool for randomized controlled trials.

Figure 1.4. 30-day complications, enhanced recovery after surgery (ERAS) protocol vs non-ERAS. A) Forest plot, and B) risk of bias assessments according to ROBINS-I tool for observational studies and C) ROB 2.0 tool for randomized controlled trials.

| Table 1 Summary of findings and Grade assessment for enhanced recovery after colorectal surgery (ERAS) compared to non-ERAS for patients with inflammatory bowel disease | | | | | | |
| --- | --- | --- | --- | --- | --- | --- |
| Outcomes | **Anticipated absolute effects^*^** (95% CI) | | Relative effect (95% CI) | № of participants (studies) | Certainty of the evidence (GRADE) | Comments |
|  | **Risk with Non-ERAS** | **Risk with ERAS** |  |  |  |  |
| Length of hospital stay | MD **1.62 days lower** (2.03 lower to 1.21 lower) | | - | 4225 (15 studies) | ⨁⨁⨁◯ Moderate^a,b^ | ERAS likely reduces length of hospital stay. |
| 30-day readmissions | 151 per 1,000 | **129 per 1,000** (99 to 166) | **OR 0.83** (0.62 to 1.12) | 4157 (15 studies) | ⨁⨁◯◯ Low^a,c^ | ERAS may result in little to no difference in 30-day readmissions. |
| 30-day reoperations | 59 per 1,000 | **41 per 1,000** (28 to 60) | **OR 0.68** (0.46 to 1.02) | 2161 (8 studies) | ⨁◯◯◯ Very low^c,d^ | The evidence is very uncertain about the effect of ERAS on 30-day reoperations. |
| 30-day complications | 296 per 1,000 | **224 per 1,000** (159 to 312) | **OR 0.69** (0.45 to 1.08) | 4074 (14 studies) | ⨁⨁◯◯ Low^a,c,e^ | ERAS may result in little to no difference in 30-day complications. |
| ***The risk in the intervention group** (and its 95% confidence interval) is based on the assumed risk in the comparison group and the **relative effect** of the intervention (and its 95% CI). **CI:** confidence interval; **MD:** mean difference; **OR:** odds ratio | | | | | | |
| **GRADE Working Group grades of evidence** **High certainty:** we are very confident that the true effect lies close to that of the estimate of the effect. **Moderate certainty:** we are moderately confident in the effect estimate: the true effect is likely to be close to the estimate of the effect, but there is a possibility that it is substantially different. **Low certainty:** our confidence in the effect estimate is limited: the true effect may be substantially different from the estimate of the effect. **Very low certainty:** we have very little confidence in the effect estimate: the true effect is likely to be substantially different from the estimate of effect. | | | | | | |

**Explanations:**

a. Downgraded one level for serious risk of bias (Effect estimate remains unchanged when serious risk of bias studies excluded. Moderate risk of bias arises primarily due inadequate statistical analysis to account for lack of randomization.)
b. Results were robust to sensitivity analysis investigating non-reporting biases and heterogeneity, so were not downgraded for these factors.
c. Downgraded one level for serious imprecision (wide confidence intervals that cross one)
d. Downgraded two levels for very serious risk of bias (all observational studies for this outcome assessed at serious risk of bias, arising primarily from inadequate statistical analysis to account for lack of randomization. The only randomized study for this outcome population size was too small with no events)
e. Heterogeneity is explained on meta-analysis of individual complications

# 2. Length of Stay (LOS)

## 2.1 Subgroup analysis. Forest plot of LOS grouped by ERAS protocol fidelity

## 2.2 Forest plot of LOS with unpublished and abstract-only data censored

## 2.3 Subgroup analysis. Forest plot of LOS grouped by inflammatory bowel disease type

## 2.4 Subgroup analysis. Forest plot for LOS grouped by studies including patients with new ostomy

## 2.5 Subgroup analysis. Forest plot for LOS grouped by studies with explicit administration of perioperative NSAIDS in ERAS arm versus studies without mention of NSAIDs

## 2.6 Subgroup analysis. Forest plot for LOS grouped by surgery type

## 2.7 Subgroup analysis. Forest plot of LOS grouped by ERAS targeted to a single surgery type versus ERAS targeted to multiple colorectal surgery types

## 2.8 Funnel plot of mean difference in LOS (days) for enhanced recovery after surgery (ERAS) protocol vs non-ERAS

## 2.9 Trimmed funnel plot of mean difference in LOS (days) for enhanced recovery after surgery (ERAS) protocol vs non-ERAS (small studies with extreme results censored)

## 2.10 Sensitivity analysis investigating non-reporting bias. Forest plot of LOS (days) for enhanced recovery after surgery (ERAS) protocol vs non-ERAS.. Small studies with extreme results censored

## 2.11 Sensitivity analysis. Forest plot of LOS using fixed effects model

## 2.12 Sensitivity analysis. Forest plot of LOS with studies at serious risk of bias censored

## 2.13 Sensitivity analysis. Forest plot of LOS, studies with potential conflicts of interest and undisclosed conflicts censored

## 2.14 Sensitivity analysis. Forest plot of LOS with potentially skewed data censored

## 2.15 Sensitivity analysis. Forest plot of LOS stratified by study design (RCT vs observational)

# 3. 30-day Readmissions

## 3.1 Subgroup analysis. Forest plot of 30-day readmissions grouped protocol fidelity

## 3.2 Subgroup analysis. Forest plot of 30-day readmissions grouped by inflammatory bowel disease type

## 3.3 Subgroup analysis. Forest plot for 30-day readmissions grouped by studies including patients with new ostomies, no new ostomies, or mixed populations (identical to 2.8)

## 3.4 Subgroup analysis. Forest plot for 30-day readmissions grouped by studies with explicit administration of perioperative NSAIDS in ERAS arm versus studies without mention of NSAIDs

## 3.5 Subgroup analysis. Forest plot of 30-day readmissions grouped by surgery type (identical to 2.8)

## 3.6 Subgroup analysis. Forest plot of 30-day readmissions grouped by ERAS targeted to a single surgery type versus ERAS targeted to multiple colorectal surgery types

## 3.7 Forest plot of 30-day readmissions using fixed effects model

## 3.8 Forest plot of 30-day readmissions reporting risk ratio

## 3.9 Forest plot of 30-day readmissions reporting risk difference

## 3.10 Forest plot of 30-day readmissions with studies at serious risk of bias censored

## 3.11 Forest plot of 30-day readmissions. Studies with potential conflicts of interest and undisclosed conflicts censored

## 3.12 Forest plot of 30-day readmissions with unpublished and abstract-only data censored

## 3.13 Sensitivity analysis 30-day readmissions stratified by study design (RCT vs observational)

## 3.14 Sensitivity analysis 30-day readmissions with alternate time frames (90 days and unspecified) censored

## 3.15 Funnel plot of 30-day readmissions for enhanced recovery after surgery (ERAS) protocol vs non-ERAS

# 4. 30-day Reoperations

## 4.1 Subgroup analysis. Forest plot of 30-day reoperations grouped by inflammatory bowel disease type

## 4.2 Subgroup analysis. Forest plot for 30-day reoperations grouped by studies including patients with new ostomies, no new ostomies, or mixed populations (identical to 3.5)

## 4.3 Subgroup analysis. Forest plot for 30-day reoperations grouped by studies with explicit administration of perioperative NSAIDS in ERAS arm versus studies without mention of NSAIDs

## 4.4 Subgroup analysis. Forest plot for 30-day reoperations grouped by surgery type (identical to 3.5)

## 4.5 Subgroup analysis. Forest plot of 30-day reoperations grouped by ERAS targeted to a single surgery type versus ERAS targeted to multiple colorectal surgery types

## 4.6 Subgroup analysis. Forest plot of 30-day reoperations grouped by protocol fidelity

## 4.7 Sensitivity analysis. Forest plot of 30-day reoperations using fixed effects model

## 4.8 Sensitivity analysis. Forest plot of 30-day reoperations reporting risk difference

## 4.9 Sensitivity analysis. Forest plot of 30-day reoperations reporting risk ratio

## 4.10 Sensitivity analysis. Forest plot of 30-day reoperations. Studies with potential conflicts of interest and undisclosed conflicts censored

## 4.11 Sensitivity analysis. Forest plot of 30-day reoperations stratified by study design (RCT vs observational)

# 5. Total complications

## 5.1 Subgroup analysis. Forest plot for total 30-day complications grouped by protocol fidelity

## 5.2 Subgroup analysis. Forest plot for total 30-day complications grouped by studies with explicit administration of perioperative NSAIDS in ERAS arm versus studies without mention of NSAIDs

## 5.3 Subgroup analysis. Forest plot of total 30-day complications grouped by ERAS targeted to a single surgery type versus ERAS targeted to multiple colorectal surgery types

## 5.4 Subgroup analysis. Forest plot of total 30-day complications grouped by surgery type

## 5.5 Subgroup analysis. Forest plot of total 30-day complications grouped by inflammatory bowel disease type

## 5.6 Subgroup analysis. Forest plot of total 30-day complications grouped by studies including patients with new ostomies, no new ostomies, or mixed populations (identical to 4.8)

## 5.7 Sensitivity analysis. Forest plot of total 30-day complications using fixed effects model

## 5.8 Sensitivity analysis. Forest plot of total 30-day complications with unpublished and abstract-only data censored

## 5.9 Sensitivity analysis. Forest plot of total 30-day complications reporting risk ratio

## 5.10 Sensitivity analysis. Forest plot of total 30-day complications reporting risk difference

## 5.11 Sensitivity analysis. Forest plot of total 30-day complications, serious risk of bias studies censored

## 5.12 Sensitivity analysis. Forest plot of total 30-day complications, studies with potential conflicts of interest and undisclosed conflicts censored

## 5.13 Sensitivity analysis. Forest plot of total 30-day complications stratified by study design (RCT vs observational)

## 5.14 Funnel plot of total 30-day complications for enhanced recovery after surgery (ERAS) protocol vs non-ERAS

# 6. Complication subcategories

## 6.1 Forest plot 30-day mortality

## 6.2 Forest plot anastomotic leaks

## 6.3 Forest plot urinary tract infections

## 6.4 Forest plot postoperative bleeds

## 6.5 Forest plot postoperative ileus

## 6.6 Forest plot venous thromboembolic complications

## 6.7 Funnel plot anastomotic leaks

## 6.8 Funnel plot postoperative bleeds

## 6.9 Forest plot combined surgical site infections

## 6.10 Funnel plot combined surgical site infections

## 6.11 Forest plot incisional (combined superficial and deep) surgical site infections

## 6.12 Forest plot superficial incisional surgical site infections (wound infections not otherwise specified included)

## 6.13 Forest plot deep incisional surgical site infections

## 6.14 Forest plot organ/space surgical site infections

## 6.15 Subgroup analysis. Forest plot of **anastomotic leak** grouped by ERAS targeted to a single surgery type versus ERAS targeted to multiple colorectal surgery types

## 6.16 Subgroup analysis. Forest plot of **bleeding** grouped by ERAS targeted to a single surgery type versus ERAS targeted to multiple colorectal surgery types

## 6.17 Subgroup analysis. Forest plot of **ileus** grouped by ERAS targeted to a single surgery type versus ERAS targeted to multiple colorectal surgery types

## 6.18 Subgroup analysis. Forest plot of **total surgical site infections** grouped by ERAS targeted to a single surgery type versus ERAS targeted to multiple colorectal surgery types

## 6.19 Subgroup analysis. Forest plot for **anastomotic leak** grouped by studies with explicit administration of perioperative NSAIDS in ERAS arm versus studies without mention of NSAIDs

## 6.20 Subgroup analysis. Forest plot for **bleeding** grouped by studies with explicit administration of perioperative NSAIDS in ERAS arm versus studies without mention of NSAIDs

## 6.21 Subgroup analysis. Forest plot for **ileus** grouped by studies with explicit administration of perioperative NSAIDS in ERAS arm versus studies without mention of NSAIDs

## 6.22 Subgroup analysis. Forest plot for **total surgical site infections** grouped by studies with explicit administration of perioperative NSAIDS in ERAS arm versus studies without mention of NSAIDs

# Primary outcomes grouped by ERAS components (sensitivity analysis)

| Table 2. Sensitivity analysis of **length of stay** for ERAS protocols including the following items: | | | | | | |
| --- | --- | --- | --- | --- | --- | --- |
|  | N studies | ERAS total, n patients | Non-ERAS total, n patients | Mean difference, days [95%CI] | p-value | I^2^ |
| Preoperative patient education | 9 | 1619 | 1735 | -1.64 [-2.20, -1.08] | <0.001 | 75% |
| Preoperative optimization | 5 | 827 | 997 | -2.15 [-3.10, -1.19] | <0.001 | 77% |
| Prehabilitation | 1 | 33 | 50 | N/A | 0.06* | N/A |
| Nutrition assessment | 4 | 591 | 773 | -2.59 [-3.81, -1.37] | <0.001 | 81% |
| Anaemia screening | 4 | 591 | 773 | -2.59 [-3.81, -1.37] | <0.001 | 81% |
| Prevention of nausea and vomiting | 6 | 1284 | 1379 | -1.51 [-2.09, -0.93] | <0.001 | 71% |
| Selective premedication | 5 | 596 | 736 | -1.87 [-2.54, -1.20] | <0.001 | 65% |
| Prophylactic IV antibiotics | 4 | 765 | 843 | -1.86 [-2.75, -0.96] | <0.001 | 76% |
| Appropriate bowel preparation | 8 | 1103 | 1187 | -1.91 [-2.54, -1.28] | <0.001 | 72% |
| Preoperative euvolemia | 6 | 612 | 752 | -2.28 [-3.11, -1.45] | <0.001 | 77% |
| No fasting and carbohydrate drink | 9 | 979 | 1055 | -1.99 [-2.60, -1.38] | <0.001 | 69% |
| Standard anaesthetic protocol | 5 | 831 | 916 | -2.00 [-2.97, -1.04] | <0.001 | 79% |
| Intraoperative normovolemia | 6 | 612 | 752 | -2.28 [-3.11, -1.45] | <0.001 | 77% |
| Intraoperative normothermia | 6 | 853 | 884 | -2.10 [-2.94, -1.26] | <0.001 | 79% |
| MIS approach | 13 | 1860 | 1971 | -1.74 [-2.21, -1.27] | <0.001 | 69% |
| No intra-abdominal drains | 4 | 319 | 280 | -2.95 [-4.60, -1.29] | <0.001 | 86% |
| No NG tube | 9 | 1253 | 1388 | -1.90 [-2.52, -1.28] | <0.001 | 76% |
| Multimodal analgesia | 11 | 1719 | 1820 | -1.59 [-2.06, -1.12] | <0.001 | 69% |
| Thromboprophylaxis | 6 | 1312 | 1482 | -1.77 [-2.48, -1.07] | <0.001 | 78% |
| Postoperative normovolaemia | 7 | 854 | 967 | -1.99 [-2.73, -1.25] | <0.001 | 75% |
| Early urinary catheter removal | 11 | 1526 | 1694 | -1.93 [-2.49, -1.38] | <0.001 | 73% |
| Prevent hyperglycemia | 1 | 236 | 224 | -1.10 [-2.08, -0.12] | 0.03 | N/A |
| Postoperative early feeding | 13 | 1859 | 2015 | -1.74 [-2.23, -1.26] | <0.001 | 70% |
| Postoperative early mobilization | 13 | 1859 | 2015 | -1.74 [-2.23, -1.26] | <0.001 | 70% |
| All protocols | 15 | 2057 | 2168 | -1.62 [-2.03, -1.21] | <0.001 | 66% |

| Table 3. Sensitivity analysis of **30-day readmissions** for ERAS protocols including the following items: | | | | | | |
| --- | --- | --- | --- | --- | --- | --- |
|  | N studies | ERAS total, n patients | Non-ERAS total, n patients | Odds ratio [95%CI] | p-value | I^2^ |
| Preoperative patient education | 10 | 1672 | 1803 | 0.91 [0.67-1.24] | 0.57 | 45% |
| Preoperative optimization | 4 | 765 | 843 | 0.78 [0.42, 1.43] | 0.42 | 68% |
| Prehabilitation | 1 | 33 | 50 | 0.13 [0.02, 1.03] | 0.05 |  |
| Nutrition assessment | 3 | 529 | 619 | 0.73 [0.29, 1.85] | 0.50 | 78% |
| Anaemia screening | 3 | 529 | 619 | 0.73 [0.29, 1.85] | 0.50 | 78% |
| Prevention of nausea and vomiting | 7 | 1373 | 1468 | 0.91 [0.65, 1.27] | 0.59 | 45% |
| Selective premedication | 6 | 685 | 825 | 0.84 [0.50, 1.41] | 0.50 | 46% |
| Prophylactic IV antibiotics | 5 | 818 | 911 | 0.68 [0.39, 1.20] | 0.18 | 67% |
| Appropriate bowel preparation | 9 | 1156 | 1255 | 0.82 [0.54, 1.24] | 0.35 | 47% |
| Preoperative euvolemia | 7 | 665 | 820 | 0.71 [0.38, 1.33] | 0.29 | 53% |
| No fasting and carbohydrate drink | 11 | 1121 | 1212 | 0.80 [0.56, 1.12] | 0.20 | 30% |
| Standard anaesthetic protocol | 5 | 822 | 830 | 0.80 [0.43, 1.48] | 0.47 | 62% |
| Intraoperative normovolemia | 7 | 665 | 820 | 0.71 [0.38, 1.33] | 0.29 | 53% |
| Intraoperative normothermia | 8 | 989 | 1020 | 1.00 [0.67, 1.48] | 0.98 | 40% |
| MIS approach | 13 | 1842 | 1921 | 0.90 [0.69, 1.17] | 0.42 | 29% |
| No intra-abdominal drains | 4 | 319 | 280 | 0.83 [0.21, 3.23] | 0.79 | 47% |
| No NG tube | 9 | 1253 | 1388 | 0.96 [0.70, 1.33] | 0.83 | 28% |
| Multimodal analgesia | 13 | 1861 | 1977 | 0.94 [0.73, 1.21] | 0.64 | 30% |
| Thromboprophylaxis | 6 | 1303 | 1396 | 0.86 [0.56, 1.31] | 0.49 | 66% |
| Postoperative normovolaemia | 8 | 907 | 1035 | 0.77 [0.48, 1.26] | 0.31 | 50% |
| Early urinary catheter removal | 10 | 1464 | 1540 | 1.00 [0.75, 1.34] | 0.98 | 25% |
| Prevent hyperglycemia | 1 | 236 | 224 | 0.74 [0.41, 1.36] | 0.34 | N/A |
| Postoperative early feeding | 13 | 1850 | 1929 | 0.93 [0.71, 1.21] | 0.60 | 32% |
| Postoperative early mobilization | 13 | 1886 | 1950 | 0.99 [0.80, 1.22] | 0.90 | 12% |
| All protocols | 15 | 2039 | 2118 | 0.83 [0.62, 1.12] | 0.23 | 49% |

| Table 4. Sensitivity analysis of **30-day reoperations** for ERAS protocols including the following items: | | | | | | |
| --- | --- | --- | --- | --- | --- | --- |
|  | N studies | ERAS total, n patients | Non-ERAS total, n patients | Odds ratio [95%CI] | p-value | I^2^ |
| Preoperative patient education | 5 | 845 | 906 | 0.69 [0.45-1.05] | 0.08 | 0% |
| Preoperative optimization | 3 | 732 | 793 | 0.65 [0.41, 1.04] | 0.07 | 0% |
| Prehabilitation | 0 | - | - | - | - | - |
| Nutrition assessment | 2 | 496 | 569 | 0.48 [0.15, 1.57] | 0.22 | 39% |
| Anaemia screening | 2 | 496 | 569 | 0.48 [0.15, 1.57] | 0.22 | 39% |
| Prevention of nausea and vomiting | 4 | 785 | 831 | 0.62 [0.39, 0.98] | 0.04 | 0% |
| Selective premedication | 2 | 496 | 569 | 0.48 [0.15, 1.57] | 0.22 | 39% |
| Prophylactic IV antibiotics | 3 | 732 | 793 | 0.65 [0.41, 1.04] | 0.07 | 0% |
| Appropriate bowel preparation | 4 | 748 | 809 | 0.65 [0.41, 1.04] | 0.07 | 0% |
| Preoperative euvolemia | 3 | 512 | 585 | 0.48 [0.15, 1.57] | 0.22 | 39% |
| No fasting and carbohydrate drink | 6 | 879 | 888 | 0.61 [0.39, 0.95] | 0.03 | 0% |
| Standard anaesthetic protocol | 1 | 250 | 144 | 0.67 [0.37, 1.23] | 0.20 | N/A |
| Intraoperative normovolemia | 3 | 512 | 585 | 0.48 [0.15, 1.57] | 0.22 | 39% |
| Intraoperative normothermia | 4 | 565 | 623 | 0.50 [0.23, 1.09] | 0.08 | 14% |
| MIS approach | 6 | 879 | 888 | 0.61 [0.39, 0.95] | 0.03 | 0% |
| No intra-abdominal drains | 2 | 266 | 160 | 0.67 [0.37, 1.23] | 0.20 | N/A |
| No NG tube | 5 | 687 | 723 | 0.65 [0.40, 1.06] | 0.08 | 0% |
| Multimodal analgesia | 6 | 898 | 944 | 0.67 [0.44, 1.01] | 0.05 | 0% |
| Thromboprophylaxis | 2 | 496 | 569 | 0.48 [0.15, 1.57] | 0.22 | 39% |
| Postoperative normovolaemia | 5 | 801 | 847 | 0.62 [0.39, 0.98] | 0.04 | 0% |
| Early urinary catheter removal | 5 | 643 | 664 | 0.55 [0.31, 0.95] | 0.03 | 0% |
| Prevent hyperglycemia | 1 | 236 | 224 | 0.75 [0.34, 1.63] | 0.46 | N/A |
| Postoperative early feeding | 7 | 976 | 985 | 0.65 [0.43, 0.98] | 0.04 | 0% |
| Postoperative early mobilization | 7 | 976 | 985 | 0.65 [0.43, 0.98] | 0.04 | 0% |
| All protocols | 8 | 1076 | 1085 | 0.68 [0.46, 1.02] | 0.06 | 0% |

| Table 5. Sensitivity analysis of **30-day complications** for ERAS protocols including the following items: | | | | | | |
| --- | --- | --- | --- | --- | --- | --- |
|  | N studies | ERAS total, n patients | Non-ERAS total, n patients | Odds ratio [95%CI] | p-value | I^2^ |
| Preoperative patient education | 9 | 1619 | 1735 | 0.81 [0.47, 1.41] | 0.46 | 89% |
| Preoperative optimization | 5 | 827 | 997 | 0.46 [0.23, 0.91] | 0.03 | 89% |
| Prehabilitation | 1 | 33 | 50 | 0.10 [0.03, 0.33] | <0.01 | N/A |
| Nutrition assessment | 4 | 591 | 773 | 0.51 [0.24, 1.09] | 0.08 | 85% |
| Anaemia screening | 4 | 591 | 773 | 0.51 [0.24, 1.09] | 0.08 | 85% |
| Prevention of nausea and vomiting | 6 | 1284 | 1379 | 0.76 [0.36, 1.61] | 0.47 | 93% |
| Selective premedication | 5 | 596 | 736 | 0.68 [0.36, 1.28] | 0.23 | 72% |
| Prophylactic IV antibiotics | 4 | 765 | 843 | 0.50 [0.23, 1.08] | 0.08 | 91% |
| Appropriate bowel preparation | 8 | 1103 | 1187 | 0.62 [0.38, 1.02] | 0.06 | 79% |
| Preoperative euvolemia | 6 | 193 | 273 | 0.71 [0.39, 1.27] | 0.25 | 65% |
| No fasting and carbohydrate drink | 9 | 979 | 1055 | 0.63 [0.39, 1.04] | 0.07 | 76% |
| Standard anaesthetic protocol | 5 | 831 | 916 | 0.69 [0.24, 1.94] | 0.48 | 92% |
| Intraoperative normovolemia | 6 | 612 | 752 | 0.71 [0.39, 1.27] | 0.25 | 65% |
| Intraoperative normothermia | 6 | 853 | 884 | 0.78 [0.51, 1.20] | 0.26 | 66% |
| MIS approach | 12 | 1762 | 1918 | 0.72 [0.44, 1.17] | 0.18 | 86% |
| No intra-abdominal drains | 4 | 319 | 280 | 0.57 [0.18, 1.78] | 0.33 | 78% |
| No NG tube | 9 | 1253 | 1388 | 0.91 [0.53, 1.53] | 0.71 | 81% |
| Multimodal analgesia | 11 | 1719 | 1820 | 0.82 [0.50, 1.35] | 0.45 | 86% |
| Thromboprophylaxis | 6 | 1312 | 1482 | 0.76 [0.40, 1.44] | 0.41 | 90% |
| Postoperative normovolaemia | 7 | 854 | 967 | 0.64 [0.36, 1.14] | 0.13 | 82% |
| Early urinary catheter removal | 11 | 1526 | 1694 | 0.80 [0.50, 1.28] | 0.35 | 81% |
| Prevent hyperglycemia | 1 | 236 | 224 | 0.33 [0.22, 0.49] | <0.001 | N/A |
| Postoperative early feeding | 13 | 1859 | 2015 | 0.75 [0.48, 1.17] | 0.21 | 85% |
| Postoperative early mobilization | 13 | 1859 | 2015 | 0.75 [0.48, 1.17] | 0.21 | 85% |
| All protocols | 14 | 1959 | 2115 | 0.69 [0.45, 1.08] | 0.10 | 85% |
